# Treatment pattern and overall survival in esophageal cancer during a 13-year period: A nationwide cohort study of 6,354 Korean patients

**DOI:** 10.1371/journal.pone.0231456

**Published:** 2020-04-10

**Authors:** Hye-Kyung Jung, Chung Hyun Tae, Hye-Ah Lee, Hyuk Lee, Kee Don Choi, Jun Chul Park, Joong Goo Kwon, Yoon Jin Choi, Su Jin Hong, Jaekyu Sung, Woo Chul Chung, Ki Bae Kim, Seung Young Kim, Kyung Ho Song, Kyung Sik Park, Seong Woo Jeon, Byung-Wook Kim, Han Seung Ryu, Ok-Jae Lee, Gwang Ho Baik, Yong Sung Kim, Hwoon-Yong Jung, Korean College of Helicobacter and Upper Gastrointestinal Research

**Affiliations:** 1 Department of Internal Medicine, College of Medicine, Ewha Womans University, Seoul, Korea; 2 Department of Medicine, Samsung Medical Center, Sungkyunkwan University School of Medicine, Seoul, Korea; 3 Department of Gastroenterology, University of Ulsan College of Medicine, Asan Medical Center, Seoul, Korea; 4 Department of Internal Medicine Institute of Gastroenterology, Yonsei University College of Medicine, Seoul, Korea; 5 Department of Internal Medicine, Daegu Catholic University School of Medicine, Daegu, Korea; 6 Department of Internal Medicine, Seoul National University Bundang Hospital, Gyeonggi-do, Korea; 7 Digestive Disease Center and Research Institute, Soonchunhyang University College of Medicine, Bucheon, Korea; 8 Department of Internal Medicine, Chungnam National University Hospital, Chungnam National University College of Medicine, Daejeon, Korea; 9 Department of Internal Medicine, St. Vincent Hospital, The Catholic University of Korea, Suwon, Korea; 10 Department of Internal Medicine, Chungbuk National University Hospital, Cheongju, Korea; 11 Division of Gastroenterology, Department of Internal Medicine, Korea University Ansan Hospital, Seoul, Korea; 12 Division of Gastroenterology and Hepatology Department of Internal Medicine, Konyang University Hospital, Daejeon, Korea; 13 Department of Internal Medicine, Keimyung University College of Medicine, Daegu, Korea; 14 Department of Internal Medicine, School of Medicine, Kyungpook National University, Daegu, Korea; 15 Department of Internal Medicine, Incheon St. Mary’s Hospital, College of Medicine, The Catholic University of Korea, Incheon, Korea; 16 Department of Internal Medicine and Digestive Disease Research Institute, Wonkwang University School of Medicine, Iksan, Korea; 17 Department of Internal Medicine, Gyeongsang National University College of Medicine and Institute of Health Sciences, Gyeongsang National University, Jinju, Korea; 18 Department of Internal Medicine, Chuncheon Sacred Heart Hospital, Hallym University College of Medicine, Chuncheon, Korea; 19 Wonkwang Digestive Disease Research Institute, Gunpo, Korea; Baylor College of Medicine, UNITED STATES

## Abstract

Using data from the real world to solve clinical questions that cannot be answered using data from clinical trials is attracting more attention. Clinical outcomes for patients with esophageal cancer in a real-world setting might be different from data in randomized controlled trials. This study aimed to provide real world data on treatment and prognosis in Korean patients with esophageal cancer. This retrospective cancer cohort included newly diagnosed cases of esophageal cancer at 19 tertiary hospitals between January 1, 2005 and December 31, 2017. Cancer staging was defined according to the 7th edition of the American Joint Committee on Cancer criteria. We identified 6,354 patients with newly diagnosed esophageal cancer (mean age: 64.9 ± 9.0 years, 96.9% squamous cell carcinoma). The proportion of early esophageal cancer increased from 24.7% in 2005 to 37.2% in 2015 (p<0.001). Among all cases, surgery alone was 31.3%, followed by definitive concurrent chemoradiotherapy (CCRT) (27.0%), neoadjuvant therapy (12.4%), adjuvant therapy (11.1%), and endoscopic resection (5.8%). The 5-year overall survival rate was 45.7 ± 0.7%. Endoscopic resection provided similar median survival relative to surgery for stage Ia cases. Among stage II–III cases, definitive CCRT was associated with poorer survival than neoadjuvant or adjuvant therapy, although there was no survival difference between neo-adjuvant and adjuvant therapy. Early esophageal cancer is gradually becoming more common and endoscopic resection provided similar long-term survival relative to surgery. Surgery with combined therapy provided better survival in locally advanced esophageal cancer, relative to definitive CCRT.

## Introduction

Esophageal cancer is the seventh most common cancer and the sixth leading cause of cancer-related mortality. [1] However, there is geographic variation in the epidemiology of esophageal cancer, with esophageal cancer being more common in Eastern and South African countries. The two most common types of esophageal cancer are squamous cell carcinoma (SCC) and adenocarcinoma (AC), which have different etiologies. Furthermore, the incidence of esophageal SCC has decreased or remained stable, especially in Asian countries, while the incidence of AC has increased rapidly in Western countries. [2] Rapid worldwide changes in cancer incidence and mortality are also occurring, which are related to changes in the prevalence and distribution of cancer risk factors, such as aging, ethnic and genetic factors and lifestyle [3] which may explain the differences between Western and Asian countries.

Given the relatively low incidence of esophageal cancer, single-center studies are not sufficient to summarize the clinical performance of esophageal cancer. Recently, use of cancer registry data linking electronic health records have shown varying performance in oncological field. The nationwide Korea Central Cancer Registry (KCCR) indicates that the incidence of esophageal cancer in 2015 was 4.7 cases per 100,000 population, with a significant predominance of male patients. [4] Recently, the annual age-standardized rates (ASRs) of esophageal cancer incidence are gradually decreasing with high mortality. Interestingly, despite a decrease in the incidence of stomach cancer, the incidence of early stomach cancer is increasing in Korea, [5] and with corresponding decreases in the proportions of regional and distant cancers. [5] This is partially related to a population-based stomach cancer screening program, which was implemented as part of the National Cancer Examination Program in 2002. The introduction of the gastric cancer screening program might also influence the diagnosis of esophageal cancer, with earlier diagnoses potentially affecting the treatment selection and prognosis. Endoscopic resection for early esophageal cancer without lymphadenopathy is recommended as alternative to esophagectomy in The National Comprehensive Cancer Network (NCCN) guidelines and the Society of Thoracic Surgeons Guidelines. [6, 7] Also NCCN for locally advanced esophageal SCC without distant metastasis recommends for definitive concurrent chemoradiotherapy (CCRT), neoadjucant therapy with surgery or esophagectomy alone. [6] However, health insurance databases or the KCCR are difficult to obtain detailed information regarding cancer stage, clinical characteristics, and treatment modality with outcomes. [8] There are limited data regarding real-world clinical practice in the field of esophageal cancer, especially regarding changes in epidemiology, treatment modalities and outcomes.

We hypothesized that clinical outcomes of patients with esophageal cancer in routine clinical care differ from those in randomized controlled trials. This study aimed to identify changes in the diagnosis, treatment, and prognosis of esophageal cancer based on a real-world cancer cohort of patients who were selected using cluster sample of Korean centers during a 13-year period.

## Subjects and methods

### Study subjects

This cohort study evaluated cases of newly diagnosed esophageal cancer at 19 referral hospitals in Korea between January 1, 2005 and December 31, 2017. The present study involved a cluster sampling of 41 tertiary hospitals in each Korean region, which should provide representative data regarding esophageal cancer management in Korea. These hospitals were selected based on known volumes, data accessibility, and location to provide an even distribution across the country. Data were requested in completely de-identified form and using a set of required variables with standard definitions. The inclusion criteria were pathologically confirmed esophageal cancer cases that were extracted based on the relevant ICD-10 diagnostic codes (C150–C159). To enhance the specificity of the diagnosis, patients were only included if they visited the hospital at least three times during the first 3 months after the initial diagnosis of esophageal cancer.

**Fig 1:**
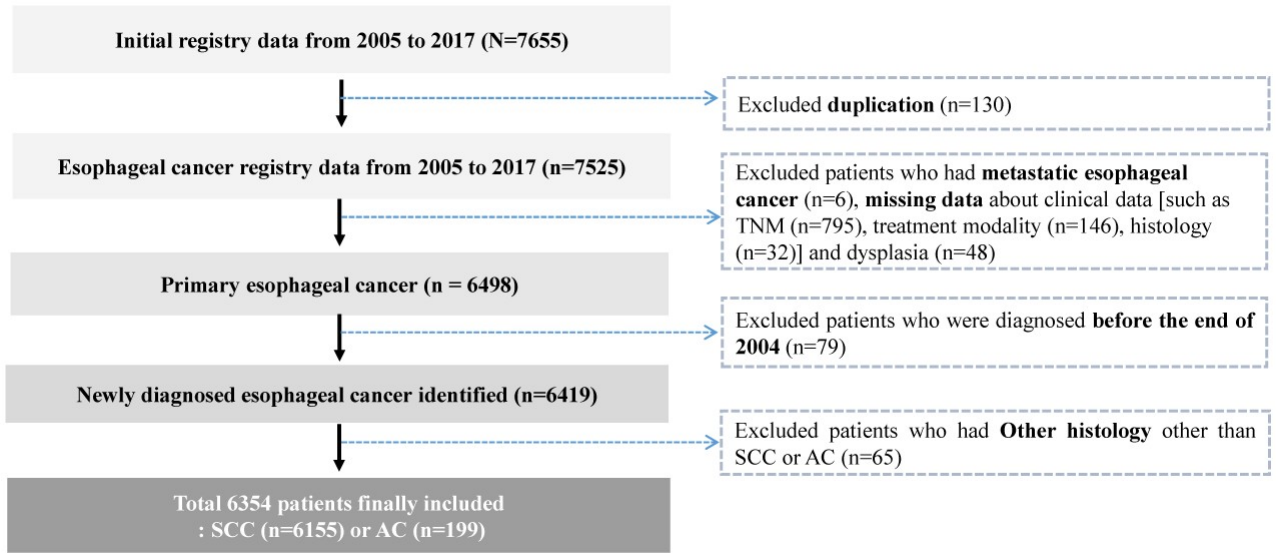
Study population. Between January 2005 and December 2017, 6,354 patients with esophageal cancer were registered. This cohort accounts for 25.8% of The Korea Central Cancer Registry, which is a nationwide hospital-based cancer registry.

A total of 7,655 patients initially fulfilled the inclusion criteria (Fig 1). However, we excluded cases based on the following criteria: 1) patients who were treated at or transferred to another hospital within 1 month (n = 130), 2) patients who had metastatic cancer or direct invasion of the esophagus (e.g., lung, thyroid, breast, or head and neck cancer) (n = 6), 3) patients with missing data regarding cancer stage (n = 795), treatment modality (n = 146), and histology (n = 32), 4) patients with dysplasia (n = 48), 5) patients with a previous diagnosis of esophageal cancer (n = 79), and 6) patients with other histological forms (non-SCC and non-AC; n = 65).

The study’s protocol was approved by all local institutional review boards, which waived the requirement for informed consent. Ewha Womans University Mokdong Hospital Institutional Review Board (2017-04-036), The institutional review board of the Samsung Medical Center, Korea (2016-11-138-001), Institutional Review Board of Asan Medical Center, Korea (2017–0079), Yonsei University Health System, Severance Hospital, Institutional Review Board (4-2016-0765), Daegu Catholic University Medical Center Institutional Review Board (CR-16-151), The institutional review board of Seoul National University Bundang Hospital (B-1611-369-108), Soonchunhyang University Bucheon Hospital Institutional Review Board (2016-09-012), Chungnam National University Hospital Institutional Review Board (CNUH 2016-11-008), Catholic Medical Center institutional review board (VC17RIME0024), Institutional review board of Chungbuk National University Hospital (CBNUH 2017-02-002-004), Korea University Ansan Hospital Institutinal Review Board (2016AS0539), Konyang University Hospital IRB (waived), Institutional review board of Keimyung University Dongsan Medical Center (2016-10-011), Kyungpook National University Chilgok Hospital Institutional Review Board (2016-11-048), Institutional Review Board of Incheon St. Mary’s Hospital (OC15QIMI0114), Wonkwang University Hospital Institutional Review Board (WKUH 201611-HR-130), Gyeongsang National University Hospital Institutional Review Board (GNUH 2016-10-005), Hallym University Chuncheon Sacred Heart Hospital Institutional Review Board (2016–115), Institutional Review Board Wonkwang University Sanbon Hospital (7302–201632). All data were obtained and analyzed anonymously, so consent was waived.

### Data collection

Data were collected regarding age, sex, height, weight, and Eastern Cooperative Oncology Group (ECOG) performance status at the initial diagnosis, as well as smoking status, alcohol consumption, comorbidities, histological subtype and grade, location, stage, treatment modality, serious complications, recurrence, and cause of death. Based on the modified criteria of Asian-Pacific guidelines of the World Health Organization, the participants were categorized according to the body mass index (BMI) as follows: normal (< 23 kg/m2), overweight (23–24.9 kg/m2) and obese (≥ 25 kg/m2). Histological subtypes (SCC and AC) and grades were assigned based on the WHO classification. [9] Anatomical location was defined as upper-third (20–25 cm from the incisors), middle-third (>25–30 cm), and lower-third cancer (>30 cm) based on the epicenter of the esophageal tumor. If multiple lesions were present, the location was defined based on the most proximal lesion. The cancer stage at the initial diagnosis was defined according to the 7^th^ edition of American Joint Committee on Cancer (AJCC) TNM staging system, [10] and was based on findings from barium esophagography, endoscopy, endoscopic ultrasonography, computed tomography, and positron emission tomography. Treatment modalities included surgical intervention, endoscopic resection, chemoradiotherapy in conjunction with surgery, CCRT, and supportive care. Endoscopic resection included endoscopic mucosal resection or endoscopic submucosal dissection under conscious sedation or general anesthesia. Surgery consisted of Ivor-Lewis esophagogastrectomy, McKeown (three-incision) esophagogastrectomy, transhiatal esophagogastrectomy, left thoracoabdominal esophagogastrectomy, and other techniques. Definitive CCRT was considered present in patients who were unfit for surgery or refused surgery and underwent chemoradiotherapy as the sole treatment with curative intent.

The date of the initial diagnosis was based on the date of the histological diagnosis. Survival outcomes were censored at December 31, 2015, and were confirmed based on a combination of active and passive approaches. Clinical follow-up data were actively checked for each hospital, and passive follow-up was performed by matching the selected cases to records from the death registry database of a national statistical organization of the Korean National Statistical Office’s death records and information regarding causes of death. All-cause mortality was assessed, and short-term mortality was defined as in-hospital mortality or mortality within 3 months after the initial diagnosis. Serious complications within 3 months were assessed using the International Conference on Harmonization Good Clinical Practice Guidelines, [11] and included adverse events that resulted in death or life-threatening events, prolonged hospitalization, and persistent or significant disability. Recurrence was defined as local recurrence, surgical wound recurrence, or distant metastasis.

### Statistical analysis

Clinical characteristics were compared using Student’s t-test or the chi-squared test, as appropriate. Kaplan-Meier curves with the log-rank test were used to compare survival according to disease stage and sub-stage classifications and Cox proportional hazard regression were used to access the survival. In order to assess the difference between the survival curves according to the overall stage (stage I, II, III and IV) and sub-staging, the log-rank test was evaluated and the *p* value was calculated using the pairwise log-rank test method to see the difference between the groups. For multiple comparisons, the adjusted *p*-values were calculated using the Bonferroni method. Factors that were associated with the overall survival (OS) outcomes were identified using a multivariate Cox proportional hazards model, with the exception of tumor stage, as the treatment modality is determined based on tumor stage. In the analysis of comparison for survival as hazard ratio (HR) according to the treatment modality, 3,486 patients out of 3510 were analyzed except patients with missing data. Among them, 2869 patients included the final analysis because of exclusion of other therapy, conservative therapy or no therapy.

In addition to sex and age, factors with a univariate *p*-value of <0.2 were considered in the multivariate analysis. Temporal survival trends were analyzed according to the year of esophageal cancer diagnosis, which was dichotomized at 2010. However, approximately one-half of the subjects in the last 2 years (2014 and 2015) were excluded because they were not monitored until 2 years after the diagnosis. Thus, we compare differences in disease stage and treatment modalities between 2005–2009 and 2010–2013. All analyses were performed using IMB SPSS software (version 25.0; IBM SPSS Statistics for Windows, Armonk, NY), and differences were considered statistically significant at *p*-values of <0.05.

## Results

### Patient characteristics

Between January 2005 and December 2017, we identified 6,354 patients who were treated at the 19 Korean centers (Fig 1). The mean age was 64.9 ± 9.0 years, and the vast majority of patients were male (92.9%) (Table 1). The esophageal cancer was diagnosed between the ages of 25 and 98 years, and was most common among patients who were 60–69 years old. The histological subtyping revealed that most cases involved SCC (96.9%), with a small proportion of AC (3.1%).

**Table 1:** Baseline characteristics of esophageal cancer.

Variables	Total (n = 6354)	SCC (n = 6155)^a^	AC (n = 199) ^b^	*P* value
Age at diagnosis (years)	64.9 ± 9.0	64.8 ± 8.9	66.7 ± 9.8	0.004
Male gender, n (%)	5902 (92.9)	5725(93.0)	177 (88.9)	0.040
BMI (kg/m^2^)	21.6 ± 3.4	21.6 ± 3.4	21.6 ± 3.7	0.906
ECOG at diagnosis^c^, n (%)				
0	3385 (57.6)	3334 (58.5)	51(27.6)	<0.001
1	2098 (35.7)	2000 (35.1)	98 (53.0)	
2	304 (5.2)	276 (4.8)	28 (15.1)	
3	65 (1.0)	60 (1.1)	5 (2.7)	
4	28 (0.5)	25 (0.4)	3 (1.6)	
Diabetes mellitus	1049 (16.6)	1011 (16.5)	38 (19.1)	0.376
Hypertension	2143 (33.8)	2083 (33.9)	60 (30.2)	0.301
Chronic renal failure	54 (0.9)	50 (0.8)	4 (2.0)	0.157
Liver cirrhosis	187 (2.9)	186 (3.0)	1 (0.5)	0.063
Ischemic heart disease	180 (2.8)	172 (2.8)	8 (4.0)	0.422
Cerebrovascular disease	202 (3.2)	187 (3.0)	15 (7.5)	0.001
Family history of esophageal cancer	106 (1.8)	102 (1.8)	4 (2.2)	0.866
Past history of other malignancy	739 (11.5)	693 (11.3)	35 (18.1)	0.012
Synchronous malignancy	337 (5.3)	259 (4.2)	78 (39.2)	<0.001
Metachronous malignancy	9 (0.1)	9 (0.1)	0	>0.99
Smoking				
Current smoker	1840 (29.0)	1776 (28.9)	64 (32.2)	<0.001
Ex-smoker	2928 (46.1)	2876 (46.7)	52 (26.1)	
Non-smoker	1365 (21.5)	1289 (20.9)	76 (38.2)	
Drinking				
Heavy alcohol drinking^d^	4469 (70.3)	4395 (71.4)	74 (37.2)	<0.001
Non-heavy drinking	1636 (25.7)	1519 (24.7)	117 (58.8)	
Unknown	249 (3.9)	241 (3.9)	8 (4.0)	

^a^SCC, squamous cell carcinoma;

^b^AC, adenocarcinoma;

^c^ECOG performance; 0, asymptomatic, 1 symptomatic, but fully ambulatory 2, in bed < 50% 3, in bed > 50%, 4 bedridden state;

^d^ Heavy drinking, defined as drinking more than 40 grams of alcohol a day for more than 10 years.

The patients were classified as having a low BMI (13.2%), having a normal BMI (51.2%), being overweight (18.1%), or being obese (17.5%). Asymptomatic patients often had an ECOG performance status of 0 (57.6%) and SCC histology (58.5%), with a smaller proportion of AC histology (27.6%). The major comorbidities were diabetes mellitus (16.6%), hypertension (33.8%), cirrhosis (2.9%), ischemic heart disease (2.8%), and cerebrovascular disease (3.2%). Some patients had a history of a malignant tumor (11.5%); lung, head and neck cancer, thyroid, or breast cancer (3.7%); and a family history of esophageal cancer (1.8%). Synchronous malignancy was detected in 5.3% of cases, and only 9 patients had metachronous lesions. Approximately 30% of patients were current smokers, and the vast majority of these patients had a smoking history of >20 years (75.1%). Heavy alcohol use was common (70.3%), which was defined as drinking >270 g/week of ethanol for >10 years.

### Staging results

**Fig 2:**
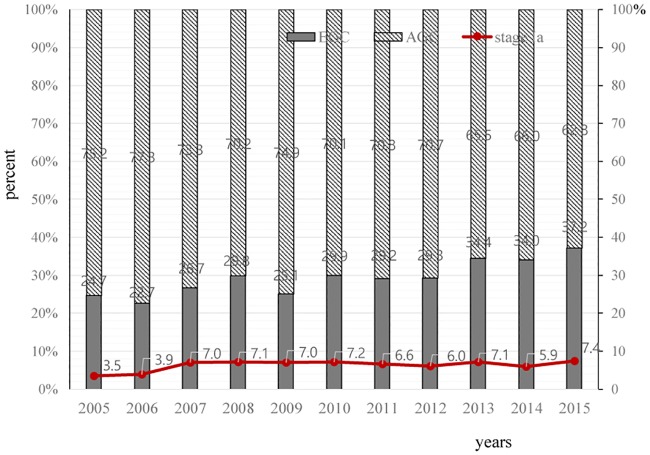
Temporal trends for early esophageal carcinoma among all esophageal cancers. The proportion of early esophageal cancers significantly increased from 24.7% in 2005 to 37.2% in 2015 (P<0.001). However, the proportion of stage Ia cases (mucosal cancer), which could be treated using endoscopy, remained relatively stable.

The AJCC 7^th^ edition staging system was used to classify cases as stage I (30.0%), stage II (28.6%), stage III (26.6%), or stage IV (14.5%) (Table 2). The temporal trend for early esophageal cancer (stage I) revealed a significant increase (from 24.7% in 2005 to 37.2% in 2015, *p*<0.001), although a relatively constant trend was observed for stage Ia disease, which can be treated using endoscopic resection (Fig 2). Histological subtyping revealed that well-differentiated tumors (grade 1) were detected in 13.5% of SCC cases and 21.1% of AC cases. Poorly differentiated tumors (grade 3) were more common in AC cases than in SCC cases (21.6% vs. 12.6%, *p*<0.001). Upper-third cancers accounted for 16.9% of cases, and lower-third cancers were the most common in SCC cases (42.3%) and AC cases (44.7%).

**Table 2:** Pretreatment clinical staging and grading of esophageal cancer.

Variables, n (%)	Total	SCC^a^	AC^b^	*P* value
cTNM Stage	N = 6354	n = 6155	n = 199	
I	1923 (30.0)	1874 (30.4)	49 (24.6)	<0.001
IA	413 (6.5)	377 (6.1)	36 (17.1)	
IB	1519 (23.0)	1497 (24.3)	13 (6.5)	
II	1820 (28.6)	1778 (28.9)	42 (21.1)	
IIA	464 (7.3)	459 (7.5)	5 (2.5)	
IIB	1356 (21.3)	1319 (214)	37 (18.6)	
III	1690 (26.6)	1632 (26.5)	58 (29.1)	
IIIA	1090 (17.2)	1055 (17.1)	35 (17.6)	
IIIB	247 (3.9)	241 (3.9)	6 (3.0)	
IIIC	353 (5.6)	336 (5.5)	17 (8.5)	
IV	921 (14.5)	871 (14.2)	50 (25.1)	
Grade (differentiation)				<0.001
G1, well	875 (13.8)	833 (13.5)	42 (21.1)	
G2, moderately	4039 (63.6)	3935 (63.9)	104 (52.3)	
G3, poorly	819 (12.9)	776 (12.6)	43 (21.6)	
Unknown	621 (9.8)	611 (9.9)	10 (5.0)	
Tumor location				<0.001
Upper	1077 (16.9)	1051 (17.1)	26 (13.1)	
Middle	2356 (37.1)	2314 (36.4)	42 (21.1)	
Lower	2692 (42.3)	2603 (42.3)	89 (44.7)	
EG junction^c^	106 (1.7)	68 (1.1)	38 (19.5)	
Unknown	123 (19.4)	119 (19.3)	4 (2.0)	

^a^SCC, squamous cell carcinoma;

^b^AC, adenocarcinoma;

^c^EG junction, esophago-gastric junction

### Treatment modalities

The vast majority of patients (93%) received some form of treatment, including palliative treatment. Among all cases, 52.3% (3,321/6,354) involved any surgical resection, which included Ivor-Lewis esophagogastrectomy (67.9%, 2,254/3,321), McKeown (three-incision) esophagogastrectomy (17.9%, 594/3,321), trans-hiatal esophagogastrectomy (2.4%, 79/3,321), left thoraco-abdominal esophago-gastrectomy (0.6%, 21/3,321), and other surgeries (11.3%, 374/3,321). The treatments included surgery alone (1,746 patients, 31.1%), endoscopic resection (323 patients, 5.8%), neoadjuvant therapy (694 patients, 12.4%), adjuvant therapy (619 patients, 11.1%), and definitive CCRT (1,717 patients, 27.0%).

Among the 1,923 patients with early-stage disease (T1N0), esophagectomy was performed in 1,096 cases (57.0%) and endoscopic resection (including endoscopic mucosal resection and endoscopic submucosal dissection) was performed in 312 cases (16.2%). Neoadjuvant and adjuvant chemotherapy were performed in 189 cases (9.8%) and definitive CCRT was performed in 164 cases (8.5%). Among patients with mucosal esophageal cancer (T1a), esophagectomy was performed in 233 cases (60.8%), which was followed by endoscopic resection (31.8%) and definitive CCRT (7.3%).

Patients with locally advanced cancer (stage II–III) often underwent combined treatment, which involved definitive CCRT or surgery with combined therapy (e.g., preoperative chemotherapy or chemoradiotherapy followed by esophagectomy; esophagectomy followed by chemotherapy or chemoradiotherapy or radiotherapy; and surgery alone). Among the 3,510 patients with stage II–III esophageal cancer, definitive CCRT was performed in 31.7%(1,133/3,510) and surgery with/without combined therapy in 54.5%(1,914/3,510) including neo-adjuvant therapy (609 patients), adjuvant therapy (518 patients), and surgery alone (788 patients). Among the 921 patients with stage IV disease, CCRT or CT or RT was performed in 47.8% (440/921) and palliative surgery was underwent in 25 cases (3.0%), an esophageal stent was inserted in 122 cases (13.2%), and percutaneous gastrostomy was performed in 11 cases (1.2%).

The treatment modalities varied significantly according to tumor location (*p*<0.001). For example, definitive CCRT was more common than surgical resection for upper-third cancers (40.5% vs. 18.5%), while surgery was more common than definitive CCRT for cancers in the lower-third or at the EG junction (33.5% vs. 22.7%). We also detected temporal changes in the treatment modalities when we compared the 2005–2009 and 2010–2013 cohorts, with significant increases noted for endoscopic resection (4.7% vs. 7.2%, *p*<0.001) and surgery with combined therapy (49.9% vs. 53.4%, *p* = 0.009), and a significant decrease for definitive CCRT (30.5% vs. 25.3%, *p*<0.001).

Serious complications developed during treatment in 537 patients (8.5%). Respiratory failure was the most common complication (256 cases, 47.3%), and was followed by fistula or other surgical site complication (108 cases, 20.2%) and sepsis or infection (81 cases, 15.0%). Serious complications were identified in 2.2% of patients who underwent endoscopic treatment, in 9.1% of patients who underwent surgery in stage I, and there was no significantly different between surgery with or without combined therapy and definitive CCRT in stage 2 (Table 3).

**Table 3:** Serious complication of esophageal cancer treatment according to the stage.

Initial treatment modality	Endoscopy (%)	Surgery ± combined therapy (%)	CCRT (%)	P value
Stage I	7/312 (2.2)	117/1285 (9.1)	15/164 (9.1)	<0.001
Stage II	0/15 (0.0)	95/1138 (8.3)	39/468 (8.3)	<0.001
Stage III	0/10 (0.0)	63/776 (8.1)	92/645 (14.3)	<0.001
Stage IV	0/0 (0.0)	10/122 (8.2)	46/440 (10.5)	<0.001

^a^, CCRT, definitive concurrent chemo-radiotherapy

After excluding the 921 patients with stage IV disease, we evaluated recurrence among 3,783 patients (69.6%) (S1 Table). We identified recurrence in 1,170 cases (30.9%), which involved the anastomosis or local recurrence (401 cases, 34.3%), regional recurrence (337 cases, 28.8%), and distant recurrence (432 cases, 36.9%). The most common site of distant metastasis was the lungs (204 cases, 17.4%), which was followed by the liver (6.3%), bone (4.0%), and multiple organs (3.1%). Later disease stages exhibited a trend towards a higher likelihood of recurrence (*p* for trend <0.0001) (S1 Table). Among patients with stage II esophageal cancer, definitive CCRT was associated with a significantly higher recurrence rate relative to surgery with combined therapy (odd ratios [OR]: 1.46, 95% confidence interval [CI]: 1.07–1.98, *p* = 0.018), although no significant difference was observed among patients with stage III disease (OR: 1.21, 95% CI: 0.90–1.64, *p* = 0.214) (Table 4).

**Table 4:** Recurrence of esophageal cancer according to the treatment.

	Total	Recurrence (%)	*P* value	Odds ratio	95% CI^a^	*P* value
Stage I	1556	267 (17.2)	0.002			
Surgery only	1187	191 (16.1)		ref.		
CCRT^b^	97	29 (29.9)		2.22	1.40–3.53	< 0.001
ER^c^	272	47 (17.3)		1.09	0.77–1.55	0.63
Stage II	1225	435 (35.5)	0.017			
Surgery and/or chemoradiotherapy	1027	350 (34.1)		ref.		
CCRT	198	85 (42.9)		1.46	1.07–1.98	0.018
Stage III	865	419 (48.4)	0.214			
Surgery and/or chemoradiotherapy	640	302 (47.2)		ref.		
CCRT	225	117 (52.0)		1.21	0.90–1.64	0.214

^a^, CI; confidence interval;

^b^CCRT, definitive concurrent chemo-radiotherapy;

^c^ER, endoscopic resection

### Overall survival

At the analysis date (December 31, 2017), we found that 3153 patients (49.6%) had died after a median observation time of 23.0 months (range: 0–146 months). The OS intervals were 9.6 years for stage I disease (95% CI: 8.9–10.3 years), 4.3 years for stage II (95% CI: 3.7–4.9 years), 1.8 years for stage III (95% CI: 1.6–2.0 years), and 0.9 years for stage IV (95% CI: 0.8–1.0 years), which were significantly different (Bonferroni-adjusted *p*-valued for all pairwise log-rank tests <0.001). The 5-year OS rate was 45.7 ± 0.7%, with rates of 72.6 ± 1.3% for stage I disease, 48.1 ± 1.4% for stage II, 29.9 ± 1.3% for stage III, and 16.6 ± 1.4% for stage IV. Increasing disease stages were associated with significantly shorter survival intervals, although we did not detect significant differences when we compared stage IIa versus stage IIb and stage IIIa versus stage IIIb.

**Fig 3:**
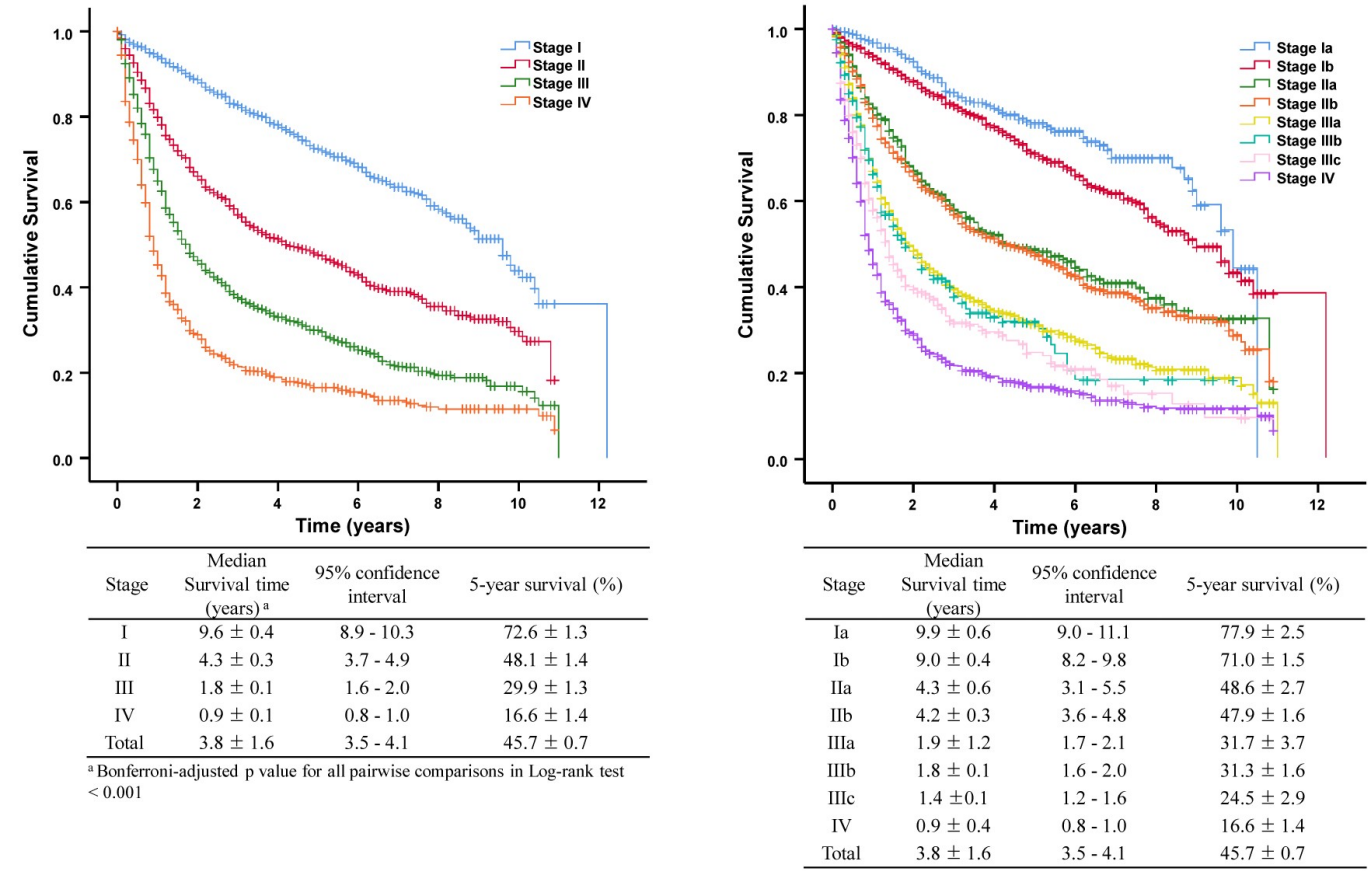
Overall survivals according to the treatment modality in early and locally advanced esophageal cancer.

Among patients with stage Ia disease, 122 of the 383 patients treated using endoscopic resection (31.9%) exhibited a similar median survival time relative to patients who underwent surgery (9.9 years vs. 10.5 years, *log-rank test p* = 0.82). Among patients with stage Ib disease, esophagectomy was associated with significantly longer OS relative to CCRT (9.8 years [95% CI: 8.2–11.4 years] vs. 4.8 years [95% CI: 3.5–6.1 years], *p*<0.001)(Fig 3). Among patients with stage II–III disease, surgery with combined therapy was associated with significantly longer OS relative to definitive CCRT (6.9 years [95% CI: 6.4–7.4 years] vs. 1.5 years [95% CI: 1.4–1.6 years], *p*<0.001). However, there was no significant difference among patients with stage IIIb or stage IIIc disease when we compared definitive CCRT and surgery with combined therapy. Among patients with stage II–III disease, significantly longer OS was associated with neo-adjuvant therapy (3.5 years, 95% CI: 2.77–4.23 years) and adjuvant therapy (3.8 years, 95% CI: 3.01–4.59 years), relative to definitive CCRT (1.6 years, 95% CI: 1.40–1.80; *p*<0.001), although no significant difference in survival was detected when we compared the neo-adjuvant and adjuvant treatment groups (Fig 3).

The multivariate Cox proportional hazards model revealed that survival was predicted by age, BMI, ECOG performance status, diabetes mellitus, liver cirrhosis, cerebrovascular accident, history of other malignancy, smoking, heavy alcohol drinking, metachronous lesions, and histological subtype (S2 Table). Among patients with stage II–III disease, definitive CCRT was independently associated with poorer survival relative to multi-modality therapy with surgery (HR: 1.75, 95% CI: 1.56–1.97, *p*<0.001) after adjusting for all prognostic factors except metachronous lesions because of the small number of cases with metachronous lesions (S3 Table).

## Discussion

This nation-wide cohort study included 6,354 patients with newly diagnosed esophageal carcinoma (predominantly SCC), which accounted for 25.8% of all South Korean esophageal cancer of KCCR over the last decade.^4^ Early esophageal cancer increased to one-third of total esophageal cancer, and treatment with endoscopic resection showed high long-term survival rate with low treatment related morbidity. And in patients with locally advanced esophageal cancer, surgery with multi-modality therapy increased OS compared to definitive CCRT, which might be different results from randomized controlled study.

We identified stage I disease in 30% of all esophageal cancer cases, which is higher than the comparable rates from Western populations, based on 17.5% (72/411) in a retrospective study at the Mayo clinic and 4.9% (70/1,417) in a prospective multicenter UK study. [12] Among them, 57% of patients with stage I disease underwent esophagectomy, and endoscopic resection was performed in 16.2%. Endoscopic resection was associated with a low rate of serious complications and a comparable long-term survival rate. However, there has been no increase in the rate of superficial mucosal esophageal cancer (Ia). It is important to clearly differentiate between stage Ia and Ib disease to determine the possibility of endoscopic treatment. In the present study, the 5-year OS rates were 77.9% for stage Ia disease and 71.0% for stage Ib disease, although this difference was not statistically significant, which limits its discriminatory value for early esophageal cancer. Endoscopic ultrasonography can be used to assess the depth of invasion, although its accuracy is only 70–88% for intra-mucosal cancers. [13, 14] Therefore, in order to increase the accuracy of cTNM classification for stage I cases, it would be useful to identify biomarkers, such as differentially expressed genes using transfer-regulatory network analysis, or to develop better endoscopy methods, such as the flexible photographing color enhancement technique. [15, 16]

The present study revealed that surgery with multimodality therapy provided significantly better survival than definitive CCRT for locally advanced cancer, even after adjustment for potential confounding factors (e.g., stage, location, histological type, performance status, and comorbidities). Esophagectomy with multi-modality therapy was performed in 79.8% of patients with stage II-III, which showed better OS, compared to other country. The 5-year survival rate in patients with stage II–III disease was 39.4%, with better survival observed for neo-adjuvant or adjuvant therapy, relative to definitive CCRT. Allum et al. also reported that neoadjuvant therapy provided better outcomes than surgery alone for locally advanced esophageal cancer, based on 5-year survival rates of 23.0% and 17.1%. [17] In the European and Japanese guidelines, perioperative chemotherapy is recommended for locally advanced AC and neo-adjuvant chemotherapy is recommended for locally advanced SCC, [18] and definitive CCRT revealed similar survival rates. [19, 20] However, in Taiwan Cancer Registry data, esophagectomy with neoadjuvant therapy showed better survival than definitive CCRT. [21] In this real world data, the proportion of patient undergoing CCRT in stage II-III rose from 17.1% to 43.0%, but the clinical outcomes was disappointing and worse than documented in previous studies. [19, 22, 23] These findings are compatible to those of our study. Data from randomized trials are usually better than observational studies, which may be due to the inclusion of healthier selected patients. Definitive CCRT is more likely to be selected for patients who are unfit for surgery, which may explain the relatively good outcomes that were associated with surgery with combined therapy. In the present study, 88.3% of all patients were treated in 5 hospitals that treated >200 cases, and 70.3% of all patients were treated in 2 hospitals that treated >2,000 cases (Samsung Medical Center and Asan Medical Center). It is highly likely that the concentration of surgical treatment for esophageal cancer in these centers was related to the good surgical outcomes.

There are limited data comparing neo-adjuvant therapy to definitive CCRT and adjuvant therapy. Neo-adjuvant therapy is expected to improve the resection rate and long-term survival by reducing the primary lesion’s size and regulating regional metastasis, and it may be possible to evaluate the patient’s response to chemotherapy or chemoradiotherapy during the histopathological examination. However, there is debate regarding the use of neo-adjuvant therapy, based on the induction of drug resistance and delayed local control, which can facilitate metastatic spreading and complicate the planned surgical procedure. [24] Randomized controlled trials are needed to compare definitive CCRT, neo-adjuvant therapy, and adjuvant therapy in high-volume centers with good surgical experience.

The present study has several strengths. First, the present study involved a cluster sampling of tertiary hospitals in each Korean region, which should provide representative data regarding esophageal cancer management in Korea. The KCCR database uses SEER staging data and does not provide detailed information including staging, treatments and clinical outcomes. The 6,354 cases from the present study account for 25.8% of KCCR registrants, [8] and the present study provided accurate data regarding long-term outcomes by integrating the Korean National Statistical Office’s death records. Second, the present study collected data from 19 hospitals, with 70% of the patients being treated at two large centralized hospitals, which presumably have relatively good surgical outcomes.

The present study also has several limitations. First, the cluster sampling was conducted among Korea’s 41 referral hospitals, and excluded primary and secondary medical institutions. Korean citizens are automatically covered by the compulsory National Health Insurance system, which provides easy and low cost access to tertiary medical services. Furthermore, given the severity of esophageal cancer, primary and secondary institutions are likely to refer patients to tertiary institutions. Therefore, cluster sampling conducted using tertiary hospitals might not significantly influence the representativeness of the findings. Second, the 19 included hospitals have varying diagnostic algorithms and therapeutic protocols, which suggests that misclassification of disease stage or treatment method is likely to affect the outcomes. Lastly, we evaluated OR instead of HR in the analysis of recurrence of esophageal cancer according to the treatment modality.

In conclusion, early esophageal cancer accounts for an increasing proportion of all esophageal cancer in Korea, while endoscopic resection provided similar long-term survival compared to surgery in early cancer. Surgery with multi-modality therapy are increasingly selected for patients with locally advanced esophageal cancer (>50% of patients), and provide a better survival that is comparable to that of definitive CCRT.

## Supporting information

S1 Table Recurrence types of esophageal cancer. (DOCX)

S2 Table Univariable Cox model analysis for prognostic factors of survival of esophageal cancer. (DOCX)

S3 Table Comparison for survival as hazard ratio according to the treatment by multivariate Cox regression model in in patients with stage II-III of esophageal cancer. (DOCX)

S1 Data (XLSX)

## Abbreviations

AC: adenocarcinoma
AJCC: American Joint Committee on Cancer
ASR: age-standardized rate
BMI: body mass index
CCRT: concurrent chemoradiotherapy
CI: confidence interval
ECOG: Eastern Cooperative Oncology Group
KCCR: Korea Central Cancer Registry
OR: odds ratio
OS: overall survival
SCC: squamous cell carcinoma
