# Supplementary material for: Treatment pattern and overall survival in esophageal cancer during a 13-year period: A nationwide cohort study of 6,354 Korean patients
Source: PLoS One. 2020 Apr 10;15(4):e0231456. doi: 10.1371/journal.pone.0231456 (PMC7147737; doi:10.1371/journal.pone.0231456)
Supplement: S1 Table — (DOCX) [file pone.0231456.s001.docx]

Supplement Table 1. Recurrence types of esophageal cancer

| Recurrence type | N (%) |
| --- | --- |
| Anastomosis / local recurrence | 401 (34.3) |
| Regional recurrence | 337 (28.7) |
| Distant | 432 (36.9) |
| Lung | 204 (17.4) |
| Liver | 74 (6.3) |
| Bone | 47 (4.0) |
| Others | 69 (5.9) |
| Multiple organ | 38 (3.1) |
