# Supplementary material for: Treatment pattern and overall survival in esophageal cancer during a 13-year period: A nationwide cohort study of 6,354 Korean patients
Source: PLoS One. 2020 Apr 10;15(4):e0231456. doi: 10.1371/journal.pone.0231456 (PMC7147737; doi:10.1371/journal.pone.0231456)
Supplement: S2 Table — (DOCX) [file pone.0231456.s002.docx]

Supplement Table 2. Univariable Cox model analysis for prognostic factors of survival of esophageal cancer

|  | HR | 95% confidence interval | *P* value |
| --- | --- | --- | --- |
| Age^a^ | 1.02 | 1.02 - 1.03 | < 0.001 |
| Male gender | 1.10 | 0.96 - 1.27 | 0.185 |
| Body mass index ^b^ | 0.93 | 0.92 - 0.94 | < 0.001 |
| ECOG (vs. 0)^c^ |  |  | < 0.001 |
| 1 | 1.62 | 1.50 - 1.74 | < 0.001 |
| 2 | 3.27 | 2.49 - 4.29 | < 0.001 |
| 3 | 4.33 | 2.89 - 6.48 | < 0.001 |
| 4 | 1.96 | 1.70 - 2.27 | < 0.001 |
| Diabetes mellitus | 1.12 | 1.02 – 1.23 | 0.015 |
| Hypertension | 0.99 | 0.92-1.07 | 0.819 |
| Cerebrovascular disease | 1.33 | 1.12-1.60 | 0.002 |
| Chronic renal failure | 1.10 | 0.76-1.59 | 0.608 |
| Liver cirrhosis | 1.38 | 1.15 – 1.65 | < 0.001 |
| Family history of esophageal cancer | 0.79 | 0.58 - 1.06 | 0.115 |
| Heavy alcohol drinking | 1.12 | 1.04 - 1.22 | 0.003 |
| Current smoking | 1.23 | 1.14 - 1.33 | 0.004 |
| Squamous cell cancer  (vs. adenocarcinoma) | 1.24 | 1.00 - 1.53 | 0.049 |
| Past history of other malignancy | 1.22 | 1.07 - 1.39 | 0.003 |
| Synchronous cancer | 1.00 | 0.86 – 1.15 | 0.950 |
| Metachronous cancer | 3.44 | 1.64 – 7.21 | 0.001 |

^a^ per 1 year; ^b^ per 1kg/m^2^; ^c^ECOG performance; 0, asymptomatic, 1 symptomatic, but fully ambulatory 2, in bed < 50% 3, in bed > 50%, 4 bedridden state; HR, hazard ratio, CI, confidence interval.
