# Supplementary material for: Treatment pattern and overall survival in esophageal cancer during a 13-year period: A nationwide cohort study of 6,354 Korean patients
Source: PLoS One. 2020 Apr 10;15(4):e0231456. doi: 10.1371/journal.pone.0231456 (PMC7147737; doi:10.1371/journal.pone.0231456)
Supplement: S3 Table — (DOCX) [file pone.0231456.s003.docx]

Supplement Table 3. Comparison for survival as hazard ratio according to the treatment by multivariate Cox regression model in in patients with stage II-III of esophageal cancer

| Treatment modality | Crude model | | | Adjusted model^a^ | | |
| --- | --- | --- | --- | --- | --- | --- |
|  | HR | 95% CI | *P* value | HR | 95% CI | *P* value |
| CCRT (n=950) vs.  Surgery with/without combined therapy | 1.96 | 1.78 - 2.16 | <0.001 | 1.75 | 1.56 - 1.97 | <0.001 |
| CCRT vs.  Neo-adjuvant therapy^b^ | 1.62 | 1.42 - 1.85 | <0.001 | 1.60 | 1.38 - 1.86 | <0.001 |
| CCRT vs. Adjuvant therapy^c^ | 1.76 | 1.53 - 2.02 | <0.001 | 1.84 | 1.55 - 2.18 | <0.001 |
| Neo-adjuvant vs. Adjuvant therapy | 1.08 | 0.92 - 1.28 | 0.340 | 1.10 | 0.91-1.34 | 0.321 |

HR; hazard ratio, CI; confidence interval, CCRT; definitive concurrent chemo-radiotherapy

^a^Adjusted for age, sex, body mass index, ECOG performance status, diabetes mellitus, liver cirrhosis, cerebrovascular disease, past history of any malignancy, smoking, alcohol, histological subtypes and tumor location; ^b^Neo-adjuvant therapy(n=629) including neo-adjuvant CCRT(n=532), neo-adjuvant CT (N=78), neo-adjuvant RT(n=19); ^c^Adjuvant therapy (n=156) including adjuvant CCRT (n=91), adjuvant CT (n=38), adjuvant RT (n=27).
